# Carrier dynamics in (Ga,In)(Sb,Bi)/GaSb quantum wells for laser applications in the mid-infrared spectral range

**DOI:** 10.1038/s41598-022-16966-x

**Published:** 2022-07-28

**Authors:** E. Rogowicz, J. Kopaczek, M. P. Polak, O. Delorme, L. Cerutti, E. Tournié, J.-B. Rodriguez, R. Kudrawiec, M. Syperek

**Affiliations:** 1 Department of Experimental Physics, Faculty of Fundamental Problems of Technology, Wrocław University of Science and Technology, Wyb. Wyspiańskiego 27, 50-370 Wrocław, Poland; 2 Department of Semiconductor Materials Engineering, Faculty of Fundamental Problems of Technology, Wrocław University of Science and Technology, Wyb. Wyspiańskiego 27, 50-370 Wrocław, Poland; 3 Department of Materials Science and Engineering, University of Wisconsin-Madison, Madison, WI 53706-1595 USA; 4 IES, CNRS, University of Montpellier, 34000 Montpellier, France

**Keywords:** Electronic devices, Applied physics, Condensed-matter physics, Semiconductors

## Abstract

We present experimental studies on low-temperature ($T={4.2}\hbox { K}$) carrier dynamics in (Ga,In)(Sb,Bi)/GaSb quantum wells (QWs) with the nominal In content of 3.7% and the Bi ranging from 6 to 8%. The photoreflectance experiment revealed the QW bandgap evolution with $-{33}\pm {1}\hbox { meV}/\hbox {at}$ % Bi, which resulted in the bandgap tunability roughly between 629 and ${578}\hbox { meV}$, setting up the photon emission wavelength between 1.97 and ${2.2}\,\upmu \hbox {m}$. The photoluminescence experiment showed a relatively small 3–10$\hbox { meV}$ Stokes shift regarding the fundamental QW absorption edge, indicating the exciton localisation beneath the QW mobility edge. The localised state’s distribution, being the origin of the PL, determined carrier dynamics in the QWs probed directly by the time-resolved photoluminescence and transient reflectivity. The intraband carrier relaxation time to the QW ground state, following the non-resonant excitation, occurred within 3–25$\hbox { ps}$ and was nearly independent of the Bi content. However, the interband relaxation showed a strong time dispersion across the PL emission band and ranging nearly between 150 and ${950}\hbox { ps}$, indicating the carrier transfer among the localised state’s distribution. Furthermore, the estimated linear dispersion variation parameter significantly decreased from $\Delta \tau \approx {20}$ to ${10}\hbox { ps}/\hbox {meV}$ with increasing the Bi content, manifested the increasing role of the non-radiative recombination processes with Bi in the QWs.

## Introduction

Bismuth-containing III–V semiconductors have aroused curiosity due to their unique physical properties, including flexibility in the bandgap tuning^1–4^ and enhanced spin-orbit splitting^5–7^. The incorporation of a small amount of Bi into the GaSb matrix reduces the bandgap of host material significantly by $\sim$30 to ${35}\hbox { meV}$/at $\%$ Bi^3^. It makes Ga(Sb,Bi)/GaSb heterostructures promising for application in optoelectronic devices theoretically operating in the 2–5$\,\upmu \hbox {m}$ spectral range, which is meaningful for environmental^8,9^, medical diagnostics^10,11^, free-space communication^12^, and spectroscopy^13^.

Especially, the bandgap tunablity, reduced Auger losses through increased spin-orbit interaction, and the type-I band alignment makes the Ga(Sb,Bi)/GaSb quantum well (QW) interesting for the active part of the mid-infrared lasers^14^. Actually, in 2017 the first GaSb$_\text {0.885}$Bi$_\text {0.115}$/GaSb QW laser was reported operating at room temperature with ${2.7}\,\upmu \hbox {m}$ emission wavelength^15^. Despite that, there are difficulties for making good quality Ga(Sb,Bi) material. The Bi atom has a large size, and therefore, it is difficult to incorporate into the III–V compounds without deteriorating their structural and optical quality. Notably, incorporation of Bi beyond the 0.12-mole fraction to the GaSb matrix allows for shifting the bandgap above ${3}\,\upmu \hbox {m}$, but causes Bi segregation, forming Bi droplets, and defects^16,17^. All these have an impact on the optical properties of the material.

In this context, the addition of In atoms can make possible it to overpass the limitations of Ga(Sb,Bi)-based structures. Gładysiewicz et al. theoretically estimated that the 8 nm-thick (Ga,In)(Sb,Bi)/GaSb QW with 32% In and 8% Bi is sufficient to achieve emission above ${3}\,\upmu \hbox {m}$^14^. Furthermore, the addition of In to Ga(Sb,Bi) should also further extend the lattice mismatch compared to GaSb, because both InSb and InBi have a bigger lattice parameter than GaSb. This can be used to increase the strain in the QW layer, which in turn would enhance the optical gain of a laser diode^18^. Moreover, Linhart et al. suggested that the In-containing material system compared to In-free owns better optical quality by substantial reduction of carrier localisation^19^. Despite promising results, knowledge about (Ga,In)(Sb,Bi)/GaSb QWs is minimal, and their optical properties, carrier dynamics are far from being fully understood^14,18–21^. In this work, (Ga,In)(Sb,Bi)/GaSb QWs with an In-content of 3.7% and various Bi-contents (see Samples growth in the "Methods" section) were studied with complementary spectroscopy techniques: the photoreflectance (PR), excitation-power dependent (PL), time-resolved photoluminescence (TRPL), and two-colour pump-probe transient reflectivity (TR).

## Results and discussion

### Steady-state spectroscopic experiments

**Figure 1:**
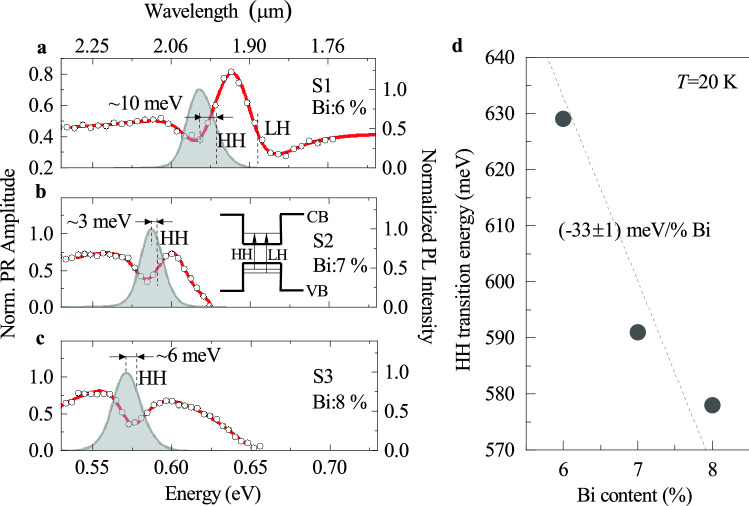
(Colour online) Low temperature ($T={20} \hbox {K}$) photoreflectance (PR) (black open circles) together with the fitting curves to Eq. (1) (solid red lines) and photoluminescence spectra (PL) (gray shaded area) for the (Ga, In)(Sb, Bi)/GaSb quantum wells (QWs) containing: (**a**) 6%, (**b**) 7%, and (**c**) 8% of Bi. The PL is measured at CW excitation with $E_\text {exc}\approx {2.33}\hbox { eV}$ and $P_\text {exc}\approx {1.13}\hbox { W}\,\hbox {cm}^{-2}$. Inset in (**b**) shows a sketch of a QW with conduction (CB) and valence bands (VB), arrows indicate absorption-like optical transitions for heavy-hole (HH) and light-hole (LH) excitons. Vertical dashed lines in (**a**)–(**c**) indicate the spectral position of the PL peak, and the HH and/or LH excitonic transition as obtained from the fit to PR data. (**d**) Evolution of the QW bandgap (HH-transition) with the Bi content. The dashed black line is a linear fit reflecting the bandgap changes at Bi%.

Figure 1a–c show low-temperature PR and PL spectra for the (Ga, In)(Sb, Bi)/GaSb QW structures S1, S2, and S3 with the Bi content of 6%, 7%, and 8%, respectively. Since the estimated electron-hole binding energy (neutral exciton binding energy) in the considered QWs is roughly ${4}\hbox { meV}$ and the charged exciton binding energy is less then ${0.7}\hbox { meV}$ (see Supplementary Information, S2), the low-temperature spectra (for $T={20}\hbox { K}$, $k_\text {B}T\approx {1.7}\,\hbox {meV}$) are largely determined by neutral excitonic features.

The PR spectrum presented in Fig. 1a for the lowest-Bi-content structure is contributed by two inhomogeneously broadened absorption-like optical features involving two fundamental exciton states confined in the QW: the heavy-hole-like (HH) and the light-hole-like one (LH) (see the sketch in Fig. 1b). For the rest of the structures, PR traces displayed in Fig. 1b and c, revealed only the HH-like exciton transition with the unresolved LH-related one. The lack of LH-related PR feature would possibly arise from the limitation of the photo-modulation mechanism connected with higher Bi content in the QWs. The PR traces are fitted by the Aspnes formula:^22^

1 $$\begin{aligned} \frac{\Delta R}{R}(E)={\text {Re}}\left[ \sum ^\text {n} _{\text {j}=1}C_\text {j}e ^{i\theta _\text {j}}(E-E_\text {j}+i\Gamma _\text {j })^{-m_\text {j}}\right] , \end{aligned}$$

where, $C_\text {j}$ is the amplitude of a PR resonance for the HH-like ($\text {j=HH}$) or LH-like excitonic transition ($\text {j=LH}$), $\text {n}$ is the number of involved transitions, $\theta _\text {j}$ is the PR phase, $E_\text {j}$ is the transition energy, and $\Gamma _\text {j}$ is the broadening parameter. The term $m_\text {j}$ is assumed to be 2, which corresponds to the PR lineshape resulted from the first derivative of a dielectric function upon photo-modulation^23^. The relevant parameters are summarized in Table 1.

**Table 1:** Extracted parameters from the PR spectra using Eq. (1) presented as a function of the Bi content in a quantum well.

Structure	Bi content	$E_\text {HH}$	$E_\text {LH}$	$\Gamma _\text {HH}$	$\Gamma _\text {LH}$	PL peak	FWHM	Stokes shift
	$\%$	(meV)	(meV)	(meV)	(meV)	(meV)	(meV)	(meV)
S1	6	629±3	653±2	22±2	28±2	620	20	10±3
S2	7	591±1	–	12±1	–	587	17	3±1
S3	8	578±1	–	13±2	–	571	20	6±1

The PL peak energy and its FWHM is derived from the Gaussian fit to the PL emission band measured at $P_\text {exc}={1.13}\,\hbox {W}\,\hbox {cm}^{-2}$.

The analysis yielded that the basic HH-exciton-like optical transition in the QWs is settled at the energy of ${\sim 629}\,\hbox {meV}$, ${\sim 589}\,\hbox {meV}$, and ${\sim 578}\,\hbox {meV}$ for the nominal Bi content of 6%, 7%, and 8% in the QW, respectively. These transition energies are plotted in Fig. 1d, following the trend predicted previously for Ga(Sb,Bi) alloys with the varied Bi content^3^. For the ${15}\,\hbox {nm}$-wide QWs, the bandgap evolves with the Bi content as ${-33}\pm {1}\,\hbox {meV}$/at % Bi, according to the linear fitting function to the experimental data (dashed black line in Fig. 1d).

The PR resonance broadening parameter $\Gamma _\text {HH}$ and the full-width-at-half-maximum (FWHM) of the PL band, included in Table 1, reflect inhomogeneities in the QW confining potential across the probed area of the sample (${\sim 150}\,\upmu \hbox {m}$ in diameter). These include QW width fluctuations, variation in the chemical content, and strain field inhomogeneities. The FWHM remains similar between examined structures when obtained at the same excitation conditions. Therefore, it would suggest that incorporating Bi doesn’t influence the spectral redistribution of radiative states within the density of states (DOS) located in the vicinity of the HH-like transition in a QW. However, increasing the Bi content increases the number of non-radiative states that affect the PL intensity causing its decrease with Bi (see Fig. 3a–c). The different nature of PR and PL experiments can explain the difference in the broadening of the PR feature and FWHM. The PL tests the occupation of DOS that depends on the excitation condition, whereas the PR tests the absorption at the available DOS. Moreover, the PR can effectively filters out the DOS related to a strongly localized states (0D states), as for which the modulation mechanism is not as effective and the DOS can be of the orders of magnitude smaller as for the extended states (2D states)^24^. It could lead to a smaller PR feature broadening then in the case of the PL. In addition, the $\Gamma _\text {HH}$ for structures with 7$\%$ and 8$\%$ of the Bi content suffers significant uncertainty related to a smaller magnitude of the PR resonance with respect to the QW with the 6$\%$ Bi content.

Additional information on the studied QWs can be derived from the comparison of PR and PL results. Figure 1 show the PL spectra (grey shaded area) obtained at $T={20}\,\hbox {K}$ and $P_\text {exc}={1.13}\,\hbox {W}\,\hbox {cm}^{-2}$. Each of the spectra was fitted by a Gaussian lineshape to extract the PL peak energy and the PL FWHM. The fitting parameters are summarized in Table 1. One can see that the PL peak energy for all the QWs is lower than the respective fundamental HH-exciton-like transition from the PR experiment. The occurrence of a Stokes shift suggests that the PL comes from localisation of excitons in the QWs. Therefore, the Stokes shift can be interpreted as the mean localisation energy. The exciton localisation for the S1 structure is nearly ${10}\,\hbox {meV}$, while for the S2 and S3 it is estimated to nearly ${3}\,\hbox {meV}$ and ${6}\,\hbox {meV}$. It is worth noting, that the Stokes shift energy for the QWs is relatively small as compared to that reported for other alloys and quantum wells belonging to the wide family of highly mismatched alloys and their heterostructures, e.g.: 14-43$\,\hbox {meV}$ for Ga(As,N) alloys^25^, 60-100$\,\hbox {meV}$ for Ga(As,Bi)/GaAs QWs^26^, ${\sim 60}\,\hbox {meV}$ for Ga(N,As,P) alloy^27^, ${\sim 120}\,\hbox {meV}$ for Ga(As,Bi)^28^, ${\sim 50}\,\hbox {meV}$ for Ga(N,P)^29^. Therefore, one can conclude that exciton localisation is weak in the studied QWs. Similar conclusion has been derived recently for the exciton localisation scenario in Ga(Sb,Bi)/GaSb QWs^30,31^, suggesting that the Ga(Sb,Bi)-based compounds behave more like a regular alloys providing better perspective for the device applications^30^.

**Figure 2:**
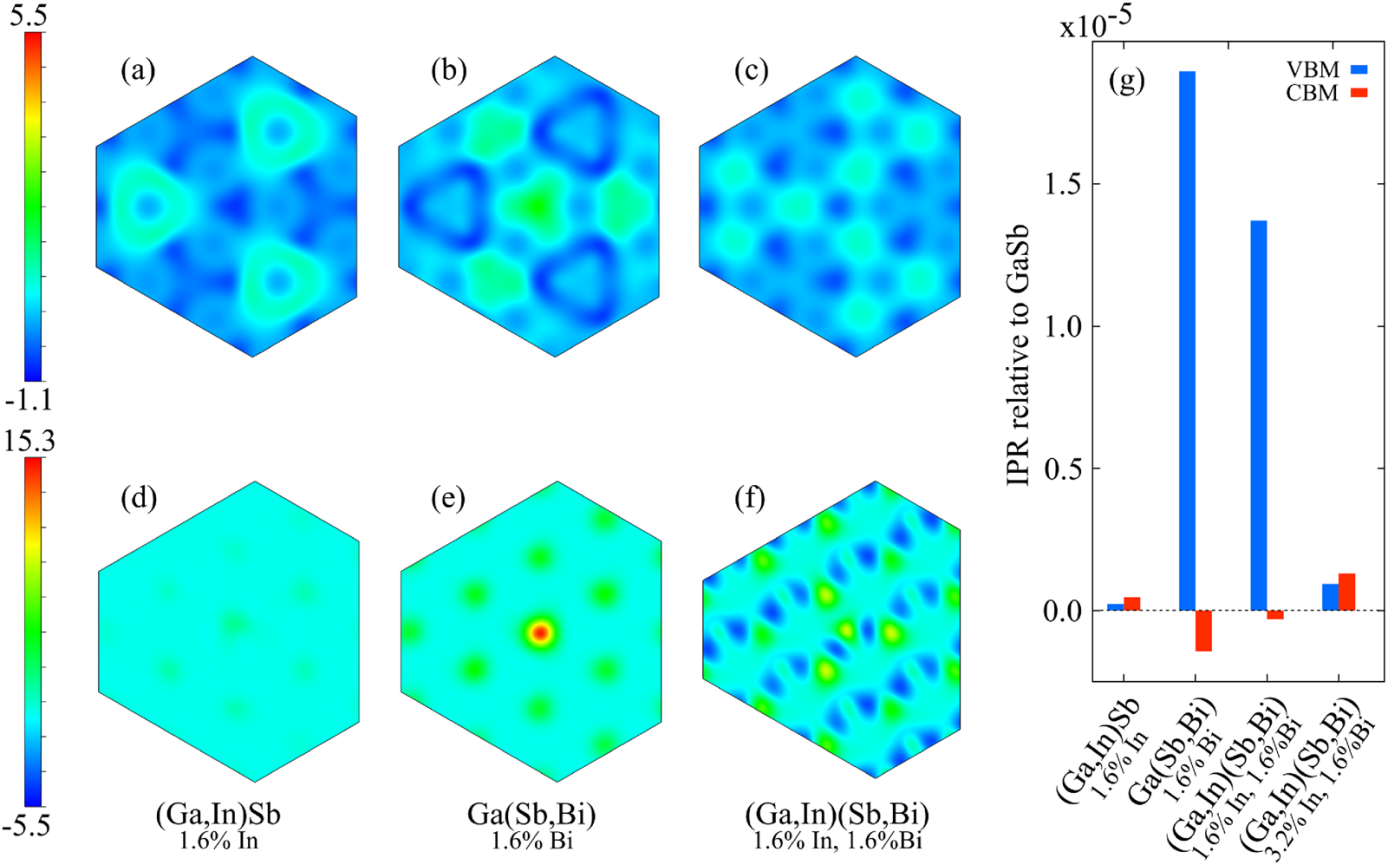
Electron localisation, presented as a difference in partial charge densities. Panels show a 2D cross section along the 111 plane, which crosses through the closest vicinity of the substituted atoms, located at the center of the unit cell. Top row (panels **a**–**c**) correspond to the conduction band minimum (CBM) of (Ga,In)Sb, Ga(Sb,Bi), and (Ga,In)(Sb,Bi) respectively. Bottom row (panels **d**–**f**) corresponds to the valence band maximum (VBM) for the same materials. The unit of the scale is $10^{-11}$. Panel (**g**) shows inverse participation ratio (IPR) relative to that of GaSb for VBM and CBM as a function of composition.

In order to gain a deeper understanding of the effects of alloying on carrier localisation, first-principles density functional theory calculations were performed. In order to analyse the localisation effects caused by alloying, we calculated real-space representations of the DFT pseudo-wavefunctions at the conduction band maximum (CBM) and valence band maximum (VBM), i.e. at the $\Gamma$ point of the Brillouin zone, for pure GaSb, Ga$_\text {1-y}$In$_\text {y}$Sb ($\text {y}=1.6\%$), GaSb$_\text {1-x}$Bi$_\text {x}$ ($\text {x}=1.6\%$), and Ga$_\text {1-y}$In$_\text {y}$Sb$_\text {1-x}$Bi$_\text {x}$ ($\text {x,y}=1.6\%$). Then, the change in the electron localisation in the alloy was calculated as the difference between obtained alloy’s partial charge density and that of pure GaSb. The 1.6% In and Bi compositions were achieved by replacing one atom in a 128-atom supercell, a $4\times 4\times 4$ multiplication of a primitive zincblende unit cell, to allow for a more straightforward analysis. Figure 2 shows the results for all three cases, with panels **a**–**c** corresponding to the conduction band minimum and panels **d**–**f** to the valence band maximum. The introduction of bismuth or indium into GaSb results in significant change in carrier localisation. The localisation associated with the CBM (Fig. 2a–c) is particularly interesting. When Bi is introduced the partial charge density of states at and close to the bottom of the conduction band is being redistributed towards the Bi atoms and its closest Sb neighbors, as can be seen in Fig. 2a. On the other hand, the introduction of indium into GaSb, has the opposite effect, where the partial charge density at and close to the VBM is redistributed away from the indium atom to neighboring Ga atoms (Fig. 2b). As a consequence, when both In and Bi are introduced, their opposite effects on charge redistribution at the conduction band edges partially cancel each other out, resulting in an overall diminished localisation and a uniform redistribution of the wave functions, which can be clearly seen in Fig. 2c. The effect of Bi on the VBM is slightly different, where a majority of the localisation and charge redistribution occurs around the Bi atom (Fig. 2d), while the presence of an indium atom in GaSb has very little effect (Fig. 2e). However, when both Bi and In are present (Fig. 2f) the localisation around the Bi atom is greatly reduced with only a slight increase in the localisation around In. In addition, the wavefunction is significantly redistributed within the bulk part of the alloy, this effect is uniform throughout the system, therefore should not have significant consequences in the optical processes. The visual representation, however, allows only for a qualitative assessment of the localization and charge redistribution, and is especially convenient when only a single atom of a given species is replaced within the unit cell, while it becomes more ambiguous for higher compositions. Therefore, for another way of evaluating localization we employed the inverse participation ratio (IPR)^32,33^, a measure that allows to quantify the localization effects based on the spatial distribution of partial charge densities. In our case, it is particularly useful to discuss the IPR relative to pure GaSb, so that only changes introduced by the allying atoms can be analyzed. The relative IPR corresponding to calculations presented in Fig. 2(a)–(f) can be found in Fig. 2(g), with an additional value for a higher In composition (3.2%). The obtained values of relative IPR for a 1.6% composition of Ga(Sb,Bi), (Ga,In)Sb and (Ga,In)(Sb,Bi) are similar to the conclusions drawn from Fig. 2: the IPR is significantly higher for the VBM when Bi is introduced and IPR for CBM is slightly lowered, while for the introduction of In, the IPR of both band edges barely change. While Bi is present, the introduction of In significantly lowers the IPR of VBM only slightly raising that of CBM. Further increase in In content continues the trend, reducing the change in IPR of VBM relative to that of GaSb to almost zero, again only slightly increasing the IPR of CBM. It is important to notice that these findings are based off of an ideal, theoretically calculated system, and only include the aspects associated with alloying, and do not take into account other effects such as the presence of defects and other crystal imperfections, which influence the localisation as well. However, the behavior presented here is likely to be one of the mechanisms contributing to the mitigation of localisation in quaternary (Ga,In)(Sb,Bi) in comparison to ternary Ga(Sb,Bi) and (Ga,In)Sb.

The PL FWHM parameters are pretty similar for all the QWs, in the range of 14–20$\,\hbox {meV}$ depending on $P_\text {exc}$. The FWHM reflects the spread in localisation energies additionally affected by the spread in a fundamental HH-exciton energy for a given QW, as discussed in the previous paragraphs.

**Figure 3:**
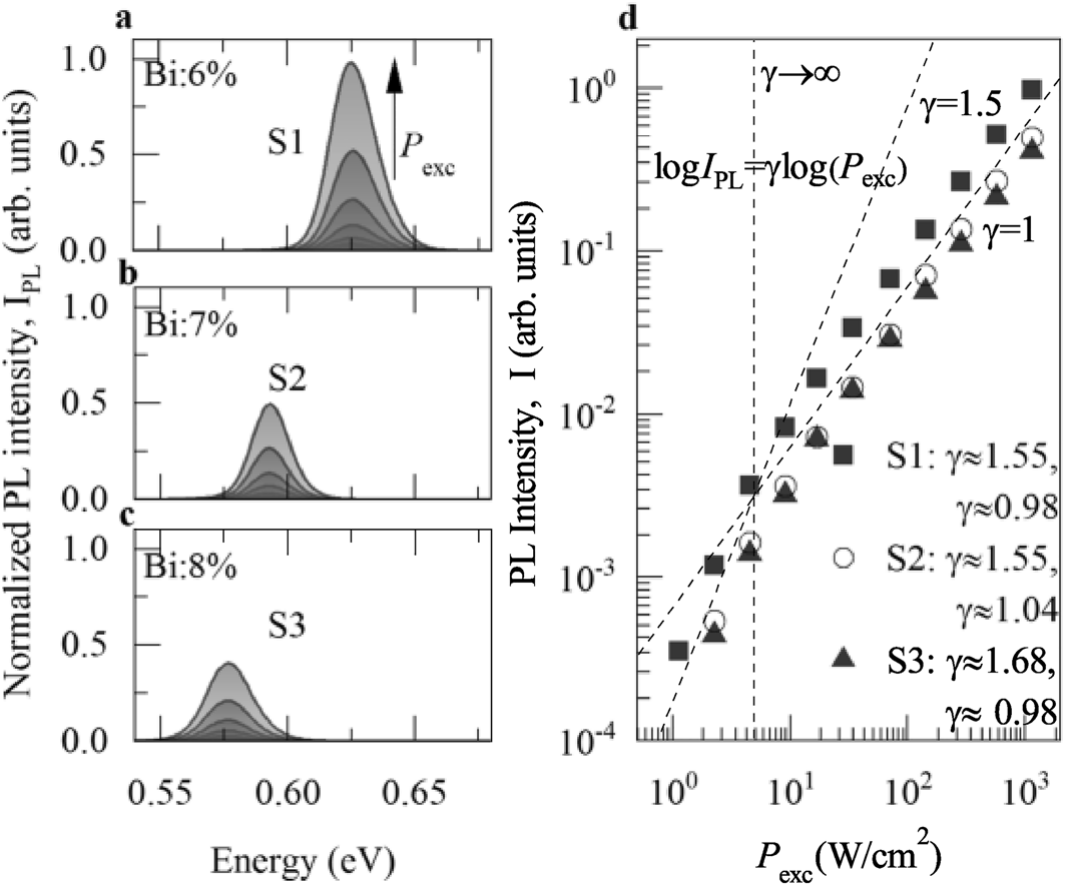
Low-temperature ($T={20}\,\hbox {K}$) power-dependent photoluminescence (PL) experiment on (Ga,In)(Sb,Bi)/GaSb QWs near the fundamental transition. (**a**)–(**c**) Evolution of PL spectra with the pumping power density ($P_\text {exc}$) for the S1, S2, and S3 structure. (**d**) Power-law function dependence for the integrated PL intensity ($I_\text {PL}$) plotted in the log-log scale (points). Black dashed lines show expected evolution of the $\log {I_\text {PL}[\log (P_\text {exc})]}$ with $\gamma \rightarrow \infty$, $\gamma =1.5$, $\gamma =1$. Legend contains the extracted $\gamma$ parameter after fitting data points with the power-law function. Experiment employed the continuous-wave excitation at $E_\text {exc}\approx {2.33}\,\hbox {eV}$. The $P_\text {exc}$ ranges from $\approx {1.13}$ to ${1132}\,\hbox {W}\,\hbox {cm}^{-2}$.

The exciton localisation scenario at low temperatures is additionally confirmed by the power-dependent PL experiment. Figures 3a–c present time-integrated PL intensity ($I_\text {PL}$) versus various continuous-wave laser excitation power density $P_\text {exc}$, ranging from ${\sim 1.13}$ to ${\sim 1132}\,\hbox {W}\,\hbox {cm}^{-2}$. The resultant $I_\text {PL}(P_\text {exc})$ function for the investigated structures is plotted in the log-log scale in Fig. 3b (open and closed points). Experimental points are fitted using the well known power-law function $I_\text {PL}\propto P_\text {exc}^{\gamma }$ to extract the $\gamma$ parameter displayed in the legend of Fig. 3d. The power-law function is well described for the studied structures in two distinguishable regimes of excitation power density. At low $P_\text {exc}$, up to several Wcm$^{-2}$, the $\gamma$ is close to 1.5, but with increasing $P_\text {exc}$ the $\gamma$ is about 1. These $\log (I_\text {PL})=\gamma \log (P_\text {exc})$ trends are visualised in Fig. 3d (dashed black lines). According to Ref.^34^, the $\gamma$ depends on the type of recombination mechanism. When the PL emission band is contributed mainly by the free exciton recombination, then $\gamma \approx {1}$. When the $I_\text {PL}$ is dominated by the defect states emission, $\gamma <1$. However, for $1<\gamma <2$, the recombination process is dictated by the bound-exciton recombination. Therefore, in the low excitation regime one can conclude that the PL emission from the studied (Ga,In)(Sb,Bi)/GaSb QWs is mainly contributed by the localised exciton recombination, while at higher pumping power, the PL seems to be controlled by the free exciton annihilation. The conclusion can be rationalised by assuming that at high pumping power the localised emission can be saturated by the large number of photo-injected electron-hole pairs and the majority of excitons populations recombines from the fundamental QW confined state.

**Figure 4:**
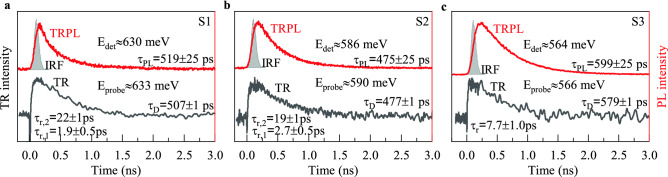
Examples of recorded low-temperature ($T={4.2}\,\hbox {K}$) time-resolved photoluminescence (TRPL) (solid red lines) and transient reflectivity (TR) traces (solid black lines) for (Ga,In)(Sb,Bi)/GaSb QWs with the nominal Bi content of (**a**) 6%, (**b**) 7%, and (**c**) 8%. The IRF (grey shaded area) represents the instrumental response function for the TRPL setup. $E_\text {det}$ and $E_\text {probe}$ are detection energy in the TRPL experiment, and the probe pulse photon energy in the TR experiment, respectively. The excitation power density in both experiments is the same, $P_\text {pump}\approx {40}\,\hbox {W}\,\hbox {cm}^{-2}$.

### Time-resolved spectroscopic experiments

We used two complementary spectroscopic techniques to monitor carrier dynamics in the (Ga,In)(Sb,Bi)/GaSb QWs: time-resolved photoluminescence (TRPL) and transient reflectivity (TR) (see Optical measurements in the "Methods" section). Figure 4 shows examples of TRPL and TR traces for the investigated QWs recorded at a given photon emission energy ($E_\text {det}$) or probe energy ($E_\text {probe}$). The structures are non-resonantly excited at $E_\text {pump}={1.49}\,\hbox {eV}$ with the same for both experiments photo-injected carrier density $P_\text {pump}\approx {40}\,\hbox {W}\,\hbox {cm}^{-2}$, generating the initial carrier population high above the GaSb barrier edge ($E_\text {g}\approx {0.8}\,\hbox {eV}$). Thus, it allows for direct data comparison between both experiments. It is essential to note that time-resolved studies are performed at $T={4.2}\,\hbox {K}$. Since the estimated electron-hole Coulomb binding energy in a (Ga,In)(Sb,Bi)/GaSb QW is roughly ${4}\,\hbox {meV}$ (see Supplemental Information, S2), one can expect that the TRPL tests mainly Coulomb-correlated electron-hole dynamics, namely neutral exciton dynamics. The higher-order exciton complexes in the QW (negatively- or positively-charged excitons or bi-exciton) can be omitted due to the lack of intentional doping of the structures. Nevertheless, we can not entirely exclude residual doping, which can originate either from the photo-excitation or defect states. However, the weak binding energy of bi-excitons or trions (see Supplementary Information, S2) substantially limits the overall meaning of these higher-exciton complexes on the data interpretation process.

**Figure 5:**
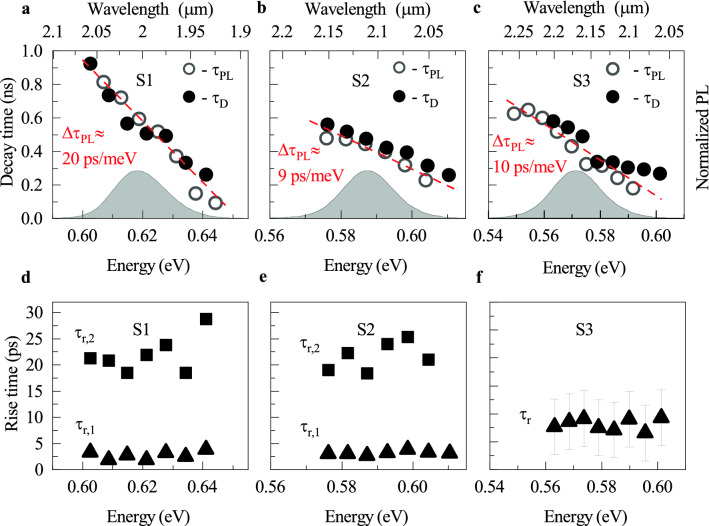
(**a**)–(**c**) Extracted decay time constants from TRPL (open circles) and TR traces analysis (black points) for (Ga,In)(Sb,Bi)/GaSb QWs with the Bi content of 6%, 7%, and 8%, respectively. Grey shaded presents normalized PL spectrum. (**d**)–(**f**) Extracted rise time constants from the TR curve analysis. $T={4.2}\,\hbox {K}$.

The typical TRPL traces for each of the QW structure S1–S3 is represented by a single-exponential rise followed by a mono-exponential decay of the PL intensity. However, due to the relatively low time resolution of the TRPL setup (${\sim 80}\,\hbox {ps}$), reflected in the full-width-at-half maximum of the instrumental response function (IRF) [a grey shaded area in Fig. 4a–c], further analysis is limited to the PL decay. It is worth noting that a similar character of TRPL traces is observed for other $E_\text {det}$ across the time-integrated PL band displayed in Fig. 1. Each of the traces is fitted to an exponential decay function $I_\text {PL}(t)\propto \exp {(t/\tau _\text {PL})}$ to extract the PL lifetime ($\tau _\text {PL}$) summarised in Fig. 5a–c.

The TR analysis provides additional information within the initial time interval of ${\sim 200}\,\hbox {ps}$ after the photo-excitation due to the much higher time resolution than in the TRPL (${\sim 300}\,\hbox {fs}$)(see Optical measurements in the "Methods" section). Within this time range, the TR amplitude evolution can be approximated by a bi-exponential rise. At a longer time scale, the TR signal undergoes a mono-exponential decay as for the TRPL. The observation holds for TR traces registered at different $E_\text {probe}$ scanned across the emission band for the QW structures S1 and S2. However, for the S3 structure, the TR traces were noisy. Therefore, just after the photo-excitation, the two components of the TR amplitude rise can not be effectively resolved leading to a single effective component. Figure 5a–c (full black points) shows the extracted TR decay time, $\tau _\text {D}$, following the numerical fitting procedure of a TR trace to a mono-exponential decay function: $I_\text {TR}\propto \exp {(t/\tau _\text {D})}$, while Fig. 5d–f illustrates the extracted TR amplitude rise times ($\tau _\text {r,1}$, $\tau _\text {r,2}$, and $\tau _\text {r}$).

The discussion starts by analysing the PL decay time spectral dependence presented in Fig. 5a–c. The $\tau _\text {PL}$ follows a strong dispersion across the PL emission band. For the S1 structure, the $\tau _\text {PL}$ increases from ${\sim 93}\,\hbox {ps}$ at ${\sim 644}\,\hbox {meV}$ to ${\sim 814}\,\hbox {ps}$ at ${\sim 607}\,\hbox {meV}$. Approximating the changes by a linear function leads to a linear PL decay dispersion variation parameter: $\Delta \tau _\text {PL}\approx {20}\,\hbox {ps}/\hbox {meV}$. For the S2 and S3 structures, the increase in the PL decay is less pronounced. For the S2 structure, the $\tau _\text {PL}\approx {227}\,\hbox {ps}$ at ${\sim 603}\,\hbox {meV}$ and increases with a $\Delta \tau _\text {PL}\approx {9}\,\hbox {ps}/\hbox {meV}$ to $\sim$576 ps at $\sim$576 meV. For the S3 structure, the dispersion starts at ${\sim 179}\,\hbox {ps}$ at ${\sim 592}\,\hbox {meV}$ and climbs with $\Delta \tau _\text {PL}\approx {10}\,\hbox {ps}/\hbox {meV}$ to ${\sim 549}\,\hbox {ps}$ at ${\sim 550}\,\hbox {meV}$.

Observation of the PL decay dispersion is related to the emission of localised excitons subjected to the additional hopping process among localised states’ distribution. The exciton localisation is suggested, first, by the inhomogeneously broadened PL band (Table 1), second, by a Stokes shift between absorption and emission (Table 1), and third, by the obtained PL lifetime. The radiative lifetime for a free or nearly free exciton confined to a type-I QW made of the III–V material system, theoretically estimated^35–37^ and experimentally verified^38–40^, is varying between 10 and 50 $\hbox {ps}$. When the exciton localization occurs at low temperature, the lifetime considerably increases due to the shrinkage of the exciton relative-motion wave function that lowers the transition oscillator strength^37,41,42^. Despite exciton trapping in the local confinement potential caused by QW width fluctuations or strain and chemical content inhomogeneities, excitons can be transferred among those traps if their density is sufficiently large, supporting a small trap-to-trap distance. The weakly bonded to the trap’s potential excitons partially contribute to the high energy tail of the PL spectrum. However, at low temperatures, the exciton transfer from the weakly bonded to strongly bonded states, emitting at the low energy tail of the PL spectrum, acts as the non-radiative process for the former ones. It leads to a decrease in the PL lifetime towards the high energy part of the PL band. The process has been initially proposed for Cd(S,Se) alloys^43^, and can be directly translated onto a QW with 0D-like states spectrally distributed below its bandgap. It has been previously identified for different QW material systems, including those belonging to the highly-mismatched alloys family: (Ga,In)(As,N)/GaAs QWs^44^, (Ga,In)(As,N,Sb)/GaAs^45^, and Ga(As,Bi)/GaAs^26^.

The non-radiative exciton decay process, represented by the non-radiative decay time ($\tau _\text {NR}$), additionally contributes to the observed PL decay time dispersion. Since all the investigated structures have the same QW width, the exciton radiative lifetime ($\tau _\text {X}$) should be a sheared property among the wells. However, Fig. 5a–c show that increasing the Bi content from 6 to 7% or 8% leads to a general reduction in the PL lifetime according to $\tau _\text {PL}^{-1}(E)=\tau _\text {X}^{-1}+\tau _\text {NR}^{-1}(E)$, where *E* is the emission energy. Thus, it leads to the conclusion that the more Bi in a QW, the higher the density of non-radiative recombination centres and the higher the non-radiative recombination rate. Moreover, nearly twice the reduction in the $\Delta \tau _\text {PL}$ with the Bi content suggests that the presence of non-radiative recombination centres, like point defects, equally affects the localised excitons distribution flattening the $\tau _\text {PL}$ dispersion.

The growing role of non-radiative recombination centres with increasing Bi content may be extended to the observed PL dispersion flattening observed for the Ga(Sb,Bi)/GaSb QWs^30^. In that case, for the ${\sim 15}\,\hbox {nm}$-wide QW with the nominal Bi content of 11%, the $\Delta \tau _\text {PL}\approx {1}\,\hbox {ps}/\hbox {meV}$ with the average PL lifetime of ${\sim 150}\,\hbox {ps}$. Therefore, the PL dynamics observed here for the (Ga,In)(Sb,Bi)/GaSb QWs may change the conclusion drawn previously from the studies on carrier dynamics in Ga(Sb,Bi)/GaSb QWs, where the Bi incorporation has not been strongly linked to with the existence of non-radiative recombination centres. However, the effect may be exactly the opposite.

The TR experiment analysis confirms the results from the TRPL one, additionally expanding the knowledge on carrier relaxation in the initial time interval after the photoexcitation. Figure 5a–c (full black points) presents the TR decay time, $\tau _\text {D}$, extracted from TR traces. The $\tau _\text {D}$ follows the same trend as the $\tau _\text {PL}$. The lack of a clear fingerprint of other relaxation components contributing to the TR decay suggests that TR probes the exciton population decay at the $E_\text {probe}$, despite abilities to test a separated electrons and holes population dynamics.

The initial carrier relaxation time for the studied QWs are presented in Fig. 5d–f. It is important to note, that this time has a composite nature due to the employed non-resonant excitation scheme. The relaxation is contributed by: (i) the carrier diffusion time in the GaSb barrier, (ii) the carrier capture time to the QW, (iii) the intraband carrier relaxation time, and (iv) the possible transfer time from the QW mobility edge to the below bandgap states and between these states.

Unambiguous assignment of the extracted time constants to a specific carrier relaxation pathway is difficult because all of these processes are hardly resolved by the specific time constants value. However, the observed components, $\tau _\text {r,1}$ and $\tau _\text {r,2}$, for both the S1 and S2 structures, may give an additional hint to the interpretation. One may speculate that the fast component ${1}\,\hbox {ps}<\tau _\text {r,1}<{3}\,\hbox {ps}$ characterised by a low TR amplitude may be related to efficient carrier relaxation in the QW following all the mentioned relaxation pathways, however, the most extended component ${17}\,\hbox {ps}<\tau _\text {r,2}<{30}\,\hbox {ps}$ of a higher TR amplitude may be imprinted by carrier diffusion process in the barrier and subsequent slower carrier capture to the QW. In this discussion, the transfer time between the QW mobility edge and the localised state’s distribution seems irrelevant because it does not show any strong spectral dependence across the tested $E_\text {probe}$ range. A similar time relaxation constant, ranging from ${14}$ to ${19}\,\hbox {ps}$, has been recently reported for Ga(Sb,Bi)/GaSb QWs^30^. The lack of observation of the two relaxation components for the S3 structure is related to the noisy TR trace preventing accurate numerical fitting. So, the extracted single rise time $\tau _\text {r}$ is an effective relaxation time.

To conclude, we investigated optical properties and exciton dynamics in (Ga,In)(Sb,Bi)/GaSb quantum wells with the varied Bi content in the range of 6–8%. The Bi incorporation allowed for tuning the QW emission energy with the -33$\pm {1}\,\hbox {meV}$ at the Bi % leading to the low-temperature photon emission in the 1.97–2.2$\,\upmu \hbox {m}$ range. The comparison between absorption and emission showed negligible Stokes shift in the range of 3–10$\,\hbox {meV}$, much smaller than that previously reported for other highly mismatched alloys and their heterostructures. It suggests that, incorporation of the Bi causes weak carrier localisation in the QW, which is promising for future device applications. Nevertheless, the carrier localization determines carrier dynamics in the QWs. We measured fast carrier relaxation channel to the QW ground state with an effective relaxation time ranging from 3 to ${25}\,\hbox {ps}$, independent on the Bi fraction. However, we found that the interband relaxation showed a strong time dispersion across the PL emission band, ranging between 150 and ${950}\,\hbox {ps}$, indicating carrier transfer among the localised state’s distribution. Furthermore, the estimated linear dispersion variation parameter significantly decreased from $\Delta \tau \approx {20}$ to ${10}\,\hbox {ps}/\hbox {meV}$ when increasing the Bi content, revealing the increasing role of the non-radiative recombination processes with Bi in the QWs. Altogether this study confirms that III-Sb-Bi alloys behave like regular III–V alloys, and that high Bi contents degrades the material quality.

## Methods

### Samples growth

The Ga$_\text {1-y}$In$_\text {y}$Sb$_\text {1-x}$Bi$_\text {x}$/GaSb QW structures were grown by solid-state molecular beam epitaxy on (001) GaSb substrates using standard effusion cells for Ga, In, Bi, and a valved-cracker-cell for Sb. The growth sequence begins with a ${100}\,\hbox {nm}$-thick GaSb buffer layer directly deposited on the substrate, followed by a ${20}\,\hbox {nm}$ AlAs$_{0.08}$Sb$_{0.92}$ barrier layer and ${180}\,\hbox {nm}$ of GaSb. The subsequent active part consists of three, ${15}\,\hbox {nm}$-wide Ga$_\text {1-y}$In$_\text {y}$Sb$_\text {1-x}$Bi$_\text {x}$ QWs separated by a ${20}\,\hbox {nm}$-thick GaSb barrier. The active part was covered by ${180}\,\hbox {nm}$ of GaSb, a ${20}\,\hbox {nm}$ AlAs$_{0.08}$Sb$_{0.92}$ barrier layer and capped by a ${20}\,\hbox {nm}$-thick GaSb layer to avoid oxidation of AlAs$_{0.08}$Sb$_{0.92}$. The AlAs$_{0.08}$Sb$_{0.92}$ barrier layers aim at preventing the photoexcited carriers to escape toward the sample surface or substrate.

Three QW structures with the same nominal 3.7% In content but various Bi fraction in a QW are investigated: (i) $\text {Bi}\sim {6}\%$, (ii) $\text {Bi}\sim {7}\%$ and (iii) $\text {Bi}\sim {8}\%$. More details about the sample growth and structure can be find elsewhere^18^.

### Optical measurements

For the PR experiment, QW structures were enclosed in a helium closed-cycle refrigerator, allowing for the sample temperature control in the range of 10–300$\,\hbox {K}$. A ${150}\,\hbox {W}$ halogen-tungsten lamp was used as a broadband probe beam source. The ${532}\,\hbox {nm}$ line ($E_\text {exc}\approx {2.33}\,\hbox {e V}$) from a continuous wave yttrium-aluminium-garnet laser was employed for photo-modulation purposes. The laser was focused on a sample surface by a lens to a spot of roughly ${150}\,\upmu \hbox {m}$ in diameter, setting up an effective samples’ surface probe area in the PR experiment. The ${0.3}\,\hbox {m}$-focal-length monochromator dispersed the white light reflected off the sample. The PR spectrum is measured via the lock-in technique at the reference modulation frequency of ${280}\,\hbox {Hz}$, using a thermoelectrically cooled PbS photodiode. The same experimental configuration with the switched-off halogen lamp was used for measuring the steady-state PL spectra.

For the time-resolved spectroscopy experiments, QW structures were hold in a helium-flow optical cryostat. In the case of the non-degenerated pump-probe TR experiment, QWs were excited by a mode-locked Ti-Sapphire laser^46,47^. The laser system generates trains of ${\sim 140}\,\hbox {fs}$-long pump pluses and ${\sim 200}\,\hbox {fs}$-long probe pulses at a repetition frequency of ${76}\,\hbox {MHz}$. The probe pulse width was measured in the homemade auto-correlation setup presented in the Supplementary Information, S1. While the photon energy of the pump pulse is kept constant at $E_\text {pump}={1.49}\,\hbox {e V}$, the probe pulse energy ($E_\text {probe}$) could be tuned in the range of $\sim$0.55 to ${0.65}\,\hbox {e V}$ by passing the train of pulses through a synchronously pumped optical parametric oscillator. The time delay between coincidences of the pump and probe pulses at the sample surface is controlled by a mechanical delay line stage, providing the scan resolution of ${\sim 300}\,\hbox {fs}$. The reflected probe beam intensity was filtered by a ${0.55}\,\hbox {m}$-focal-length monochromator and measured in the lock-in scheme by the liquid-nitrogen-cooled InSb-based detector at the ${3}\,\hbox {kHz}$ carrier frequency.

The TRPL was measured with a PL setup by a time-correlated single-photon counting method. In this case, the sample was excited non-resonantly by a train of ${\sim 140}\,\hbox {fs}$-long pulses from the Ti:Sapphire oscillator with the photon energy in the pulse at $E_\text {pump}={1.49}\,\hbox {e V}$. The TRPL signal was filtered by a ${0.5}\,\hbox {m}$-long monochromator and photons were collected by the NbN superconducting detector. The overall temporal resolution of the TRPL setup was ${\sim 80}\,\hbox {ps}$^48^.

### First principles calculations

First principles calculations were carried out with the VASP software package^49,50^. Plane Augmented Wave potentials (PAW)^51^ were used, with *d* electrons treated as valence. Due to the large atomic mass of the atoms involved, spin-orbit coupling was included as well. The geometry and atomic positions were fully optimized using the PBEsol functional^52^ known for its high accuracy in calculating lattice constants of III–V and similar systems^53,54^. A 2$\times$2$\times$2 k-point Monkhorst-Pack mesh was used. In order to correct for the band gap underestimation, particularly important in the case of low band gap GaSb, the mBJLDA functional was used^55^, which has been shown numerous times to be an accurate and efficient method for band structure calculations for a wide variety of systems, including III–V semiconductors and their alloys^56–58^.

## Supplementary Information

Supplementary Information.
