# Supplementary material for: Carrier dynamics in (Ga,In)(Sb,Bi)/GaSb quantum wells for laser applications in the mid-infrared spectral range
Source: Sci Rep. 2022 Jul 28;12:12961. doi: 10.1038/s41598-022-16966-x (PMC9334638; doi:10.1038/s41598-022-16966-x)
Supplement: Supplementary file 1 — Supplementary Information. [file 41598_2022_16966_MOESM1_ESM.pdf]

Supplementary Information: Carrier dynamics in (Ga,In)(Sb,Bi)/GaSb quantum wells for laser applications in the mid-infrared spectral range

E. Rogowicz[1,*], J. Kopaczek[2], M. P. Polak[2,3], O Delorme[4], L. Cerutti[4], E. Tournié[4], J.-B. Rodriguez[4], R. Kudrawiec[2,+], and M. Syperek[1]

[1]Laboratory for Optical Spectroscopy of Nanostructures, Faculty of Fundamental Problems of Technology, Department of Experimental Physics, Wrocław University of Science and Technology, Wyb. Wyspiańskiego 27, 50-370 Wrocław, Poland
[2]Faculty of Fundamental Problems of Technology, Department of Semiconductor Materials Engineering, Wrocław University of Science and Technology, Wyb. Wyspiańskiego 27, 50-370 Wrocław, Poland
[3]Department of Materials Science and Engineering, University of Wisconsin-Madison, Madison, Wisconsin 53706-1595, USA
[4]IES, University of Montpellier, CNRS, 34000 Montpellier, France
[*]ernest.rogowicz@pwr.edu.pl
[+]robert.kudrawiec@pwr.edu.pl

ABSTRACT

We present experimental studies on low-temperature ($T = 4.2\,\text{K}$) carrier dynamics in (Ga,In)(Sb,Bi)/GaSb quantum wells (QWs) with the nominal In content of 3.7% and the Bi ranging from 6% to 8%. The photoreflectance experiment revealed the QW bandgap evolution with -33 $\pm$ 1 meV/at % Bi, which resulted in the bandgap tunability roughly between 629 meV to 578 meV, setting up the photon emission wavelength between 1.97 µm and 2.2 µm. The photoluminescence experiment showed a relatively small 3-10 meV Stokes shift regarding the fundamental QW absorption edge, indicating the exciton localisation beneath the QW mobility edge. The localised state's distribution, being the origin of the PL, determined carrier dynamics in the QWs probed directly by the time-resolved photoluminescence and transient reflectivity. The intraband carrier relaxation time to the QW ground state, following the non-resonant excitation, occurred within 3-25 ps and was nearly independent of the Bi content. However, the interband relaxation showed a strong time dispersion across the PL emission band and ranging nearly between 150 ps and 950 ps, indicating the carrier transfer among the localised state's distribution. Furthermore, the estimated linear dispersion variation parameter significantly decreased from $\Delta\tau \approx 20\,\text{ps/meV}$ to $10\,\text{ps/meV}$ with increasing the Bi content, manifested the increasing role of the non-radiative recombination processes with Bi in the QWs.

S1. Two-photon absorption autocorrelation

The laser pulse width diagnostics in the near-infrared spectral range above the 2 µm photon wavelength is very limited. Therefore, to estimate the time resolution of the pump-probe transient reflectivity experiment, we have developed a dedicated experimental setup to measure the laser probe pulse width based on the intensity autocorrelation utilizing the two-photon absorption (TPA) phenomenon in a semiconductor material. The intensity autocorrelation setup diagram is shown in Fig. S1**a**.

The laser source (in our case, the synchronously-pumped optical parametric oscillator) generates a sequence of identical pulses in the near-infrared. After leaving the source, pulses are divided by a 50%/50% beam splitter into a tested and probe pulse train. The probe pulse is directed to the retro-reflector placed on a mechanical delay line stage with a maximum travel length of 30 cm. Changing the position of the delay line offers time-varied intensity correlation between the probe and tested pulses on an InGaAs photodiode. The intensity correlation can be probed within the time range up to $\sim$2 ns, which allows checking the distance between subsequent pulses in the pulse train (if possible) with a minimum time step of $\sim$13 fs. The time step is the ultimate resolution of the autocorrelation setup. The intensity correlation between pulses is seen through the photodiode output voltage generated due to the non-linear TPA process in the active material of the photodiode. The linear response of the photodiode is limited to the 1.9 µm photon wavelength. The TPA voltage is measured, using the lock-in amplifier at the reference frequency of the mechanical modulator.

The example of the autocorellation TPA signal measured for the pulse photon wavelength of 2.2 µm and the average power $P_{\text{ave}} = 740\,\mu\text{W}$ is presented in Fig. S1**b**. The pulse profile is approximated by a Gaussian function (red line in Fig. S1**b**). The

fitting procedure allowed extracting the autocorrelation half-width, which is $\tau_{AC} = 281\,\text{fs}$. Thus the pulse width is obtained using the relation $\frac{\tau_{AC}}{\tau_{pulse}} = \sqrt{2}$, giving the value of the probe pulse length $\tau_{pulse} \approx 200\,\text{fs}$.

Fig. S1**c** shows the response of the InGaAs photodiode in the non-linear regime of its operation, where the TPA processes are present. The TPA is a third-order non-linear phenomenon, its efficiency scales non-linearly with the number of photons involved.

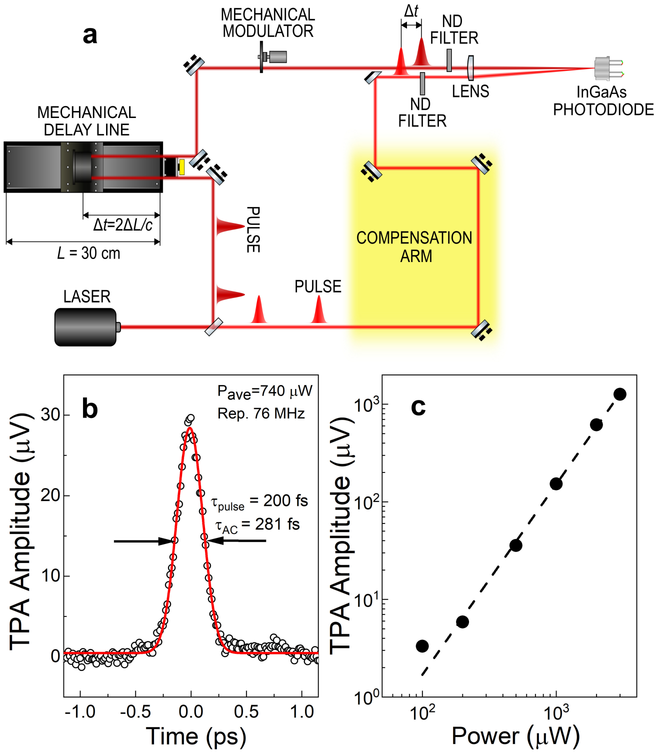

Figure S1. **a** Scheme of a setup for two-photon absorption (TPA) autocorrelation measurement. **b** Autocorrelation TPA signal for the pulse photon wavelength of 2.2 μm and the average power of $P_{ave} = 740\,\mu\text{W}$. **c** Dependence of the TPA signal on the average power of autocorrelated pulses.

S2. Theoretical estimations of the neutral and charged exciton binding energy in the antimonides-based quantum wells

The theoretical estimation is made to evaluate the neutral and charged exciton (trion) binding energies in the studied antimonide-based quantum wells (QWs). The neutral exciton binding energy ($E_{X,QW}$) is evaluated with the following formula[1]:

$$E_{X,QW} = \frac{E_X}{\left[1 - \frac{1}{2}\exp(-d_{QW}/2a_X)\right]^2}, \tag{1}$$

where E_X and a_X are the bulk exciton energy and radius, whereas d_{QW} is the QW width.

The charged-exciton binding energy ($E_{T,QW}$) is estimated using the equation[2]:

$$E_{T,QW} = \frac{E_X}{3\sqrt{\frac{d_{QW}}{a_X}}}. \tag{2}$$

The bulk exciton energy E_X and radius a_X were defined as:

$$E_X = \frac{\mu}{m_0 \varepsilon_S^2} \times 13.6\,\text{eV} \quad \text{and} \quad a_X = \frac{\varepsilon_S}{\mu} a_B, \tag{3}$$

where m_0 represents the free electron mass, ε_S is the static permittivity, a_B is the Bohr radius and μ is the reduced mass of an exciton. The μ is defined by:

$$\frac{1}{\mu} = \frac{1}{m_\mathrm{e}^{*\mathrm{GaSb_{1-x}Bi_x}}} + \frac{1}{m_\mathrm{h}^{*\mathrm{GaSb_{1-x}Bi_x}}}, \tag{4}$$

where $m_\mathrm{e}^{*GaSb_{1-x}Bix}$[3] and $m_\mathrm{h}^{*\mathrm{GaSb_{1-x}Bix}}$[3] are the effective masses of an electron and a hole, respectively. They are derived from by the following analytical formulas:

$$m_\mathrm{e}^{*\mathrm{GaSb_{1-x}Bix}} = m_\mathrm{e}^{*\mathrm{GaSb}} - 0.212x - 0.015x^2 \quad \text{and} \quad m_\mathrm{h}^{*\mathrm{GaSb}} = m_\mathrm{h}^{*\mathrm{GaSb}} - 0.01x, \tag{5}$$

where x represents the Bi fraction in GaSb, $m_\mathrm{e}^{*\mathrm{GaSb}}$ and $m_\mathrm{h}^{*\mathrm{GaSb}}$ are an electron and a hole effective masses in GaSb, respectively. We want to stress that there are no available similar equations for the (Ga,In)(Sb,Bi) material system. However, we believe these estimations based on GaSbBi will give similar results.

For the calculations, we used the following GaSb parameters: $\varepsilon_S = 15.7$, $m_\mathrm{h}^{*\mathrm{GaSb}} = 0.4m_0$, $m_\mathrm{e}^{*\mathrm{GaSb}} = 0.042m_0$[3].

The neautral exciton binding energy and the charged exciton binding energy as a function of the Bi content are summarized in Tab. 1.

Structure	Bi content %	$E_{\mathrm{X,QW}}$ (meV)	$E_{\mathrm{T,QW}}$ (meV)
S1	6	4.03	0.71
S2	7	3.84	0.68
S3	8	3.63	0.66

Table 1. Estimated exciton and trion binding energies based on Eq. 1 and 2 presented as a function of the Bi content in a quantum well.

S3. Supercells used in DFT calculations

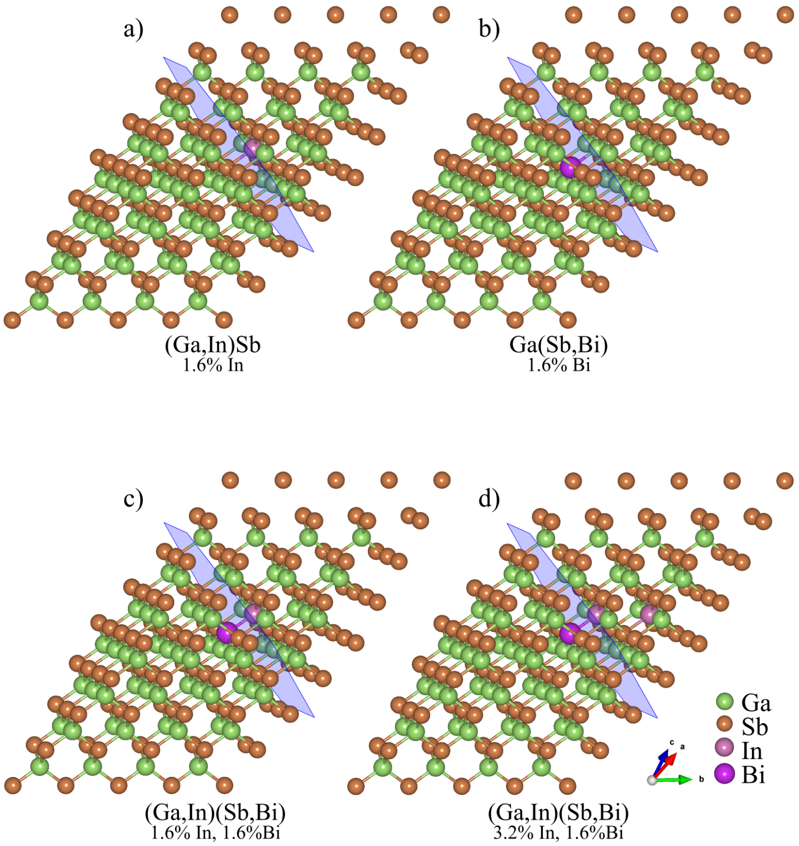

Figure S2. The supercells used in the DFT calculations. They correspond to results presented in Fig. 2. The plane on which the partial densities are projected in Fig. 2 (**a**-**f**) is indicated in blue.

References

1. Mathieu, H., Lefebvre, P. & Christol, P. Simple analytical method for calculating exciton binding energies in semiconductor quantum wells. *Phys. Rev. B* **46**, 4092–4101, DOI: 10.1103/PhysRevB.46.4092 (1992).

2. Sergeev, R. A., Suris, R. A., Astakhov, G. V., Ossau, W. & Yakovlev, D. R. Universal estimation of X- trion binding energy in semiconductor quantum wells. *The Eur. Phys. J. B - Condens. Matter Complex Syst.* **47**, 541–547, DOI: 10.1140/epjb/e2005-00362-5 (2005).

3. Ammar, I., Sfina, N. & Fnaiech, M. Optical gain and threshold current density for mid-infrared GaSbBi/GaSb quantum-well laser structure. *Mater. Sci. Eng. B* **266**, 115056, DOI: https://doi.org/10.1016/j.mseb.2021.115056 (2021).
